# Investigating links between Internet literacy, Internet use, and Internet addiction among Chinese youth and adolescents in the digital age

**DOI:** 10.3389/fpsyt.2023.1233303

**Published:** 2023-09-07

**Authors:** Qiaolei Jiang, Zonghai Chen, Zizhong Zhang, Can Zuo

**Affiliations:** ^1^School of Journalism and Communication, Tsinghua University, Beijing, China; ^2^Chinese Academy of Social Sciences (CASS), Beijing, China

**Keywords:** Internet addiction, Internet literacy, Internet use, youth and adolescents, China

## Abstract

**Introduction:**

In current digital era, adolescents’ Internet use has increased exponentially, with the Internet playing a more and more important role in their education and entertainment. However, due to the ongoing cognitive, emotion, and social development processes, youth and adolescents are more vulnerable to Internet addiction. Attention has been paid to the increased use of Internet during the COVID-19 pandemic and the influence of Internet literacy in prevention and intervention of Internet addiction.

**Methods:**

The present study proposes a conceptual model to investigate the links between Internet literacy, Internet use of different purpose and duration, and Internet addiction among Chinese youth and adolescents. In this study, *N* = 2,276 adolescents studying in primary and secondary schools in East China were recruited, and they completed self-reports on sociodemographic characteristics, Internet literacy scale, Internet use, and Internet addiction scale.

**Results:**

The results showed a significant relationship between Internet use and Internet addiction. To be specific, the duration of Internet use significantly and positively affected Internet addiction. With different dimensions of Internet literacy required, entertainment-oriented Internet use had positive impact on Internet addiction, while education-oriented Internet use exerted negative effects on Internet addiction. As for Internet literacy, knowledge and skills for Internet (positively) and Internet self-management (negatively) significantly influenced the likelihood of Internet addiction.

**Discussion:**

The findings suggest that Internet overuse increases the risk of Internet addiction in youth and adolescents, while entertainment-oriented rather than education-oriented Internet use is addictive. The role of Internet literacy is complicated, with critical Internet literacy preventing the development of Internet addiction among youth and adolescents, while functional Internet literacy increasing the risk.

## 1. Introduction

In recent years, the world has witnessed rapid development and diffusion of digital technologies, especially for the Internet. International Telecommunication Union (ITU) estimated that there were 5.3 billion Internet users in the world, accounting for approximately 66% of the global population in 2022 (1). By the end of 2022, there were 1.07 billion Internet users in China, with the penetration rate of 75.6%, of which 18.7% were Chinese netizens aged 19 and younger (2). According to the latest official statistics on adolescent Internet users, Internet users under the age of 18 in China reached 191 million in 2021, with Internet penetration as high as 96.8% of the Chinese population of youth and adolescents (3).

Nowadays, the Internet exerts a powerful influence on daily life, and it is bringing about a digital life that can be seen, touched and felt. For youth and adolescents as digital native, the Internet is so indispensable for entertainment, relaxation, interpersonal communication, information acquisition, as well as learning and education. However, as the younger generation engage in various online activities and spend a considerable amount of time online, the potential risks associated with Internet use (IU) or its adverse impacts on adolescents have attracted wide attention, including Internet addiction (IA) (4). A meta-analysis of 31 countries estimated IA prevalence rate worldwide at 6.0% (5), which was even higher in China (6).

Due to those serious social and psychological consequences of Internet addiction, increasing academic studies keep a watchful eye on the intervention of adolescent Internet addiction, which has identified many effective intervention strategies, such as cognitive behavioral therapy, family therapy, and school-based prevention programs (7–9). Although many efforts have been made to promoting media literacy among the young, studies being conducted to test the effectiveness of Internet literacy in preventing Internet addiction are far from enough. Therefore, this study attempted to explore the possible relationships between Internet literacy, Internet use, and Internet addiction among Chinese youth and adolescents, so as to provide empirical findings and practical implications for effective prevention of Internet addiction among the kids in the digital age.

## 2. Literature review

### 2.1. Internet addiction: a pressing issue among youth and adolescents in the digital age

With the development and increasing importance of Internet, the side effects have also aroused growing public concern, such as Internet addiction. In 1995, Internet addiction was initially coined by American psychiatrist Ivan Goldberg, and then empirically studied by pioneer scholars, such as Kimberly Young and Mark Griffiths. Internet addiction is defined as the incapacity to control one’s online behaviors, mainly manifested as excessive use of the Internet, leading to adverse outcomes (10).

Based on existing empirical research, there are two primary theoretical traditions of Internet addiction have been established. The first, exemplified by Kimberly Young, identifies Internet addiction as an impulse control disorder, separate from substance addiction but sharing similar features (11). The second, represented by Mark Griffiths, views Internet addiction as a form of technology addiction, belonging to behavioral addiction and encompassing all core components of behavioral addiction. Griffiths specifically emphasizes that Internet addiction results from excessive human-computer interaction prompted by the inducement and reinforcement characteristics of the medium and specific activities (12). Despite variations in theoretical conceptualization of Internet addiction, most of current studies recognize compulsivity and impairment as the two essential elements of Internet addiction (13). To be specific, compulsivity refers to the difficulty in regulating the impulse to go online and experiencing intense cravings when unable to access the Internet, while impairment relates to negative effects such as neck and back pain, insomnia, anxiety, depression, loneliness, social isolation, and poor academic performance resulting from excessive Internet use.

As for youth and adolescents, current studies on IA prevalence present high variability worldwide. The prevalence of Internet addiction in European young and adolescent community samples was approximately 4–10% (14), while it ranged from 0 to 26.3% among American undergraduate students (15). According to those studies with the focus on Asia, the overall prevalence of Internet addiction in Southeast Asian general populations was 20.0% (16), and it was estimated to be 3–26.8% in Hong Kong adolescents (17). Although these prevalence rates varies depending on the heterogeneity of measurement instruments and research samples, Internet addiction has been found to show profound negative influence on youth and adolescents. Those adolescents who suffer from Internet addiction have demanded considerable investment of time and effort online that they may have a series of physical health diseases or problems, such as obesity, eye strain, headaches, eating disorders, and lowered sleep quality (18). Apart from its adverse effects on physical health, Internet addiction also threatens the mental health of teenagers. A wealth of evidences have suggested that Internet addiction can increase the risk of psychological distress, negative emotions, body dissatisfaction, alexithymia, and less subjective well-being (19, 20).

In China, the rising prevalence of the Internet, especially mobile Internet usage has brought about a significant concern for Internet addiction in the young generation (4). A meta-analysis indicates that the prevalence of Internet addiction is growing in China, which now reaches 11.3% among Chinese younger generation (6). In order to address this pressing issue, the latest amended version of *the Law on the Protection of Minors* has added a special chapter named *Internet Protection*, which further highlights the potential risks youth and adolescents may face in cyberspace and prompts strong social attention. Correspondingly, regulation, prevention, intervention, and various strategies have been carried out to prevent excessive Internet use among Chinese minors and cope with adverse impact of Internet addiction.

### 2.2. Entertainment vs. education: two types of Internet use among youth and adolescents

According to an authoritative report, 97% of the American teenagers use the Internet daily, with video apps like YouTube and TikTok being the primary source of media consumption (21). Similarly, Chinese youth also spend quite a lot of time online. The national Internet use report among youth and adolescents indicates that 88.9 and 62.3% of teenage netizens frequently use educational and gaming applications, respectively, (3), revealing two critical motives for the young to use the Internet, i.e., education and entertainment.

Entertainment-oriented IU is characterized as a type of pleasure-seeking self-moderation *via* the Internet (22), encompassing activities such as gaming, video streaming, social media browsing, etc. Existing research has predominantly concentrated on the impact of entertainment-based IU on emotional regulation, such as reducing stress (23) and satisfying intrinsic human needs (22). During the COVID-19 pandemic, consumption of entertainment content online continued to rise, serving as coping strategies for emotional disturbance caused by the external environment (24).

Meanwhile, there is a lot of education-oriented IU among youth and adolescents, which involves utilizing applications like Zoom or Tencent Meeting for e-learning or online coursework. In recent years, the enforcement and promotion of education-oriented IU among the youth have substantially increased as a result of home quarantine policies implemented in response to the global health crisis (25). Some scholars find that urgent remote education has significantly enhanced students’ academic performance during the early stages of the COVID-19 pandemic (26).

As for the inter-relationship between Internet use and Internet addiction, past literatures have shown that prolonged Internet use is linked to higher risks of addiction, with long-term usage being identified as one of the most significant factors contributing to teenager Internet addiction (27). Nevertheless, as Young stated, the Internet itself is not addictive, while various types of Internet use, such as gaming, online chatting, social networking sites, short-form videos, and smartphone apps, are possible predictive factors for Internet addiction (11, 28). Hence, different forms of Internet use may exert dissimilar influences on Internet addiction. Although both entertainment-oriented and educational-oriented IU have played vital roles during young people’s daily life, these two types of Internet use may differ in their impact on Internet addiction among youth and adolescents. Specifically, entertainment-oriented IU, such as gaming, has been found to be closely related with adolescents’ dependence and addiction to the Internet (28). Moreover, studies have identified a surge in problematic Internet use resulting from increasing exposure to entertainment media in the COVID-19 outbreak (29). In contrast, existing studies have not reached a consensus on whether education-oriented IU will lead to Internet addiction. Some scholars contend that the proliferation of online education has increased the duration of students’ Internet use, which may further lead to Internet addiction (30), while others believe that education-oriented IU is more likely to result in cyberloafing than direct Internet addiction (31). Different from previous research, the current research aims to take both the duration and forms of Internet use into consideration, and examine their relationship between Internet use and Internet addiction among Chinese youth and adolescents. Thus, the following research question was raised.

**RQ1**: How does Internet use, including both duration and two types, i.e., entertainment-oriented and education-oriented IU, affect Internet addiction among Chinese youth and adolescents?

### 2.3. Internet literacy: a multidimensional concept

Internet literacy was coined by McClure about 30 years ago, who believe that this emerging concept encompasses the capacity to comprehend the value of online resources, utilize search tools to retrieve specific information and assist individuals in problem-solving (32). However, over the past few decades, a consensus has not yet been reached on the definition of Internet literacy within academia. According to Dupuis, Internet literacy should encompass knowledge and comprehension of the informational background in contemporary society, the composition and arrangement of information, as well as its utilization in lifelong learning (33). After Shapiro and Hughes proposed a seven-dimensional structure for Internet literacy (34), subsequent scholars have defined Internet literacy as the capability to access, understand or analyze, and generate digital content, in response to the advancement of various media technologies (35).

Internet literacy is understood and deconstructed by some scholars from two main aspects, both skill literacy and information literacy. The former pertains to the abilities related to using and incorporating the Internet, while the latter focuses on people’s ability to access, collect, and filter information (36). Given the intricate dimensions and structures of Internet literacy, it is likely that proficiency levels among youth and adolescents may vary across different aspects of Internet literacy. Particularly in the era of digital and even intelligent media technologies, there is a growing need for expertise in media consumption, while adolescents, who are often known as digital natives, tend to exhibit a superior level of Internet skill literacy (27, 37). Facing a vast amount of complex information, particularly in the post-COVID-19 era, where misinformation and disinformation still abound, it is crucial to be able to perceive information critically. With regard to teenagers, although they tend to have higher level of skill literacy, their critical thinking abilities concerning online information still need enhancement, resulting in an uneven distribution of different aspects of their Internet literacy (38).

Previous studies have investigated the link between Internet literacy and various aspects of Internet use, including IU duration and different types of IU. A study involving 2,303 students found no significant correlation between media literacy and the number of days or hours spent consuming media (39). Regarding different types of IU, a survey of 1,018 individuals by van Deursen and his colleagues showed that higher proficiency in Internet skills were associated with more Internet use for information-seeking and career-related purposes (40). Additionally, Internet literacy has been found to be a significant predictor to explain online political engagement among adolescents and enhance the capacity of individuals to participate in different types of online user interactions (41). Overall, prior studies failed to reach a consensus regarding the impact of Internet literacy on Internet use, and did not take both IU duration and types of online activity into consideration simultaneously. In light of this, this study seeks to fill the gap through investigating the impact of Internet literacy on Internet use with consideration of both use duration and types.

**RQ2:** How do different dimensions of Internet literacy influence Internet use among Chinese youth and adolescents?

### 2.4. Internet literacy and Internet addiction

Whether Internet literacy can have significant influence on prevention and intervention of Internet addiction, pertinent literatures have demonstrated a complex relationship between Internet literacy and Internet addiction, with both positive and negative effects of different aspects of Internet literacy linked to Internet addiction. For instance, a survey carried out in Germany revealed that higher levels of technical literacy may be positively associated with Internet addiction, whereas higher levels of reflective and self-regulatory skills are linked to a smaller likelihood of Internet addiction (42). In a study involving structured questionnaire interviews with 718 Hong Kong adolescents, two researchers found that certain types of Internet literacy, such as publishing and technology skills, could increase the risk of Internet addiction (27, 37). Similarly, a positive link between information and communication technology (ICT) literacy and problematic Internet use in adolescents was reported (43). On one hand, scholars from different social contexts find positive relationship between certain dimensions of Internet literacy and Internet addiction. On the other hand, some research found no significant correlation between Internet literacy and Internet addiction among teenagers (44). Therefore, it seems that the multi-dimensional structure of Internet literacy can have varied and even quite different impacts on Internet addiction, which calls for more empirical studies to reveal specific mechanism of different dimensions of Internet literacy on Internet addiction. Additionally, despite the growing number of youth netizens in contemporary China, studies on Internet literacy and Internet addiction of this group are still far from enough. Previous studies have also emphasized the cultural uniqueness of Internet addiction in China (45). Thus, the following research question was proposed to investigate the association between Internet addiction and Internet literacy in the Chinese context.

**RQ3**: As a multidimensional concept, how do different dimensions of Internet literacy influence Internet addiction among Chinese youth and adolescents?

**Figure 1:**
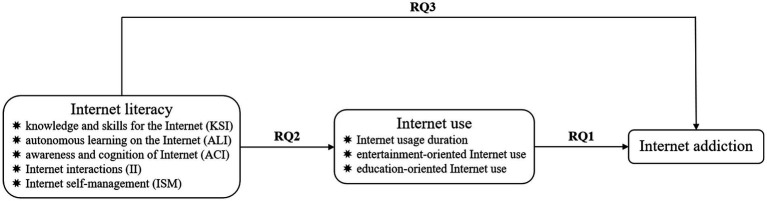
Conceptual framework of the links between Internet literacy, Internet use and Internet addiction.

Based on the aforementioned literature review on Internet addiction, Internet use and Internet literacy, the following conceptual framework (see Figure 1) was proposed to answer the three research questions, so as to investigate the links between Internet literacy, Internet use, and Internet addiction among Chinese youth and adolescents in current digital age.

## 3. Methods

### 3.1. Data collection

The survey data was collected in East China. Primary and middle students from a county-level city in Suzhou, Jiangsu Province participated in this research. With the assistance of the local education bureau, a paper-based survey was conducted in October 2022, by using random sampling and quota sampling. Firstly, according to the basic conditions of schools within the region in Suzhou Statistical Yearbook (46), specific percentages and planned sample size of students from local primary school, middle school, and high school were calculated based on student enrollment. Secondly, various situations of different types of local schools were also fully considered, and 5 primary schools, 3 middle schools, 3 high schools, 1 vocational high school, 1 nine-year school and 1 twelve-year school, therefore in total 14 schools were randomly selected for this study. Thirdly, classes of each school were numbered and the corresponding classes (30–50 students in each class) were selected by using random number table. Finally, 40 primary school classes, 20 middle school classes and 14 high school classes were selected. Given that students of first grade in primary school had just entered the nine-year compulsory education system and might not fully understand the measurement items in the questionnaire, they were excluded from the survey. Table 1 shows the sampling details.

**Table 1:** Details of the student enrollment, planned sample size and actual sample size.

School type	Student enrollment	The planned sample size	The actual sample size
Primary school	94,834 (64.1%)	1,923	1,607 (50.4%)
Middle school	37,155 (25.1%)	753	870 (27.3%)
High school	15,875 (10.8%)	324	644 (20.2%)
Total	147,864	3,000	3,187 (with 66 user-missing values)

Based on the aforementioned sampling procedures, participants were recruited with consent from the students, their parents and teachers. In total, 3,187 paper-based questionnaires were distributed and collected, and 2,276 were confirmed as valid. As for the sample profile, the participants were aged 7–18 (Mean = 12.28, SD = 2.85), 52.9% (1204) were male, and 47.1% (1072) were female. In terms of age, 434 students (52.9%) were aged 7–9, 779 (34.2%) were aged 10–12, 627 (27.5%) were aged 13–15, and 436 (19.2%) were aged 16–18. Regarding their grade levels, 1,069 (47.0%) were primary school students, 677 (29.7%) were middle school students, and 530 (23.3%) were high school students.

### 3.2. Measurement

#### 3.2.1. Internet use

Given the limitations of time-based or frequency-based measurement, Internet use was measured by both IU duration and Internet activities in this study. The two items (“How much time do you spend online everyday, from Monday to Friday?” and “How much time do you spend online everyday, on weekends or holidays?”) were measured on a five-point Likert scale (from 1 = not at all to 5 = more than 3 h) to assess IU duration among the participants (see Supplementary Appendix 1 for the results of IU duration). The reliability Cronbach’s *α* was 0.689. As for Internet activities, a five-point Likert scale (1 = not at all to 5 = always) was adopted to measure the frequency of online entertainment, information acquisition, online learning, and social interaction *via* the Internet (i.e., How often do you engage in entertainment/learning/information acquisition/social interaction through the Internet every day?). The reliability Cronbach’s α was 0.647, which was not optimal but also surpassed the acceptable level (47).

#### 3.2.2. Internet literacy

The Internet Literacy Scale (ILS) developed by Huang et al. (48), which was verified among Chinese junior students, was adopted to investigate multiple dimensions of Internet literacy in the present study. The 18-item scale (see Supplementary Appendix 2) defines the structure of Internet literacy with five main dimensions, including knowledge and skills for the Internet (KSI, e.g., I can make good use of Internet tools, such as office software and search engines), autonomous learning on the Internet (ALI, e.g., I can access and analyze useful information on the website to complete learning tasks), awareness and cognition of the Internet (ACI, e.g., I think the Internet is a double-edged sword), Internet interactions (II, e.g., I am able to make new friends through the Internet), and Internet self-management (ISM, e.g., I can control how much time I spend online). Participants were asked to respond to these items by using a five-point Likert scale (from 1 = strongly disagree to 5 = strongly agree). The reliability Cronbach’s α was remarkably high at 0.917.

#### 3.2.3. Internet addiction

The proposed diagnostic criteria for Internet addiction by Tao et al. (49) was adopted in this survey. Participants needed to answer eight items by using a five-point Likert scale (from 1 = strongly disagree to 5 = strongly agree). The reliability Cronbach’s α was 0.885.

#### 3.2.4. Demographics

The investigators collected demographic characteristics on the gender, grade and age of participants.

### 3.3. Statistical analysis

The statistical analyses were performed by using SPSS and AMOS. Firstly, exploratory factor analysis (EFA) was run to explore the multi-type Internet activities among Chinese adolescents. Secondly, the construct validity of Internet literacy in this research sample was examined through confirmatory factor analysis (CFA). Finally, this study adopted structural equation model (SEM) to examine the inter-relationships between Internet literacy, Internet use and Internet addiction.

Additionally, *a priori* power analysis by G*Power was used to estimate the sample size of SEM by assuming a small effect size of 0.02 and α of 0.05. The research model encompassed a total of 11 predictors, consisting of five independent variables (five subdimensions of Internet literacy), three mediators, and three control variables. The outcome of the G*Power analysis indicated that a minimum sample size of 1,267 participants would be necessary to achieve a desired statistical power of 95%. Actually, the current study went beyond the requirements by involving a sample of 2,276 adolescents as research participants. This sample size significantly exceeded the threshold suggested by G*Power, which further enhanced the statistical robustness of the study.

## 4. Results

### 4.1. Exploratory factor analysis of Internet use

In order to examine the multi-structure of Internet use among Chinese adolescents, an EFA was conducted. Principal component analysis was used as the method for factor extraction. The results demonstrated that the original 4 items were formed into two types of Internet use, i.e., entertainment-oriented and education-oriented IU. Based on eigenvalues greater than 1.0, EFA generated two factors that together explained 75.742% of the total variance. To be specific, the first factor, entertainment-oriented IU (eigenvalue = 1.991, 45.078% of variance, Cronbach’s α = 0.658), consisting of 2 items, revealed the respondents’ frequency of online entertainment and social interaction through the Internet. Education-oriented IU (eigenvalue = 1.039, 30.664% of variance, Cronbach’s α = 0.709) as the second factor showed the respondents’ frequency of information acquisition *via* the Internet and online learning. In addition, parallel analysis was conducted for factor extraction, and the results (see Supplementary Appendix 3) indicated that the number of factors was two according to both the mean and percentile of parallel analysis, which further supported the factor extraction based on EFA. Table 2 presents the results of EFA.

**Table 2:** Exploratory factor analysis of Internet use (IU).

Items	Mean	SD	Factors	
			1	2
1, Entertainment-oriented IU				
Entertainment	3.218	1.365	0.868	−0.023
Social interaction	2.778	1.521	0.845	0.115
2, Education-oriented IU				
Information acquisition	3.432	1.328	0.213	0.773
Online learning	3.393	1.285	−0.031	0.941
Eigenvalue			1.991	1.039
Percent of variance explained			45.078	30.664
Cronbach’s α			0.658	0.709

### 4.2. Confirmatory factor analysis of Internet literacy

A CFA was carried out to examine the construct validity of ILS. The fit indices indicated that χ^2^/df = 6.411, CFI = 0.977, GFI = 0.971, NFI = 0.973, TLI = 0.963, and RMSEA = 0.049. The Cronbach’s α was 0.917, and the composite reliability (CR) of each dimension was greater than 0.8, indicating that ILS had good internal consistency. Moreover, all the standardized factor loadings were highly significant at the 99% confidence level, and all the average variance extracted (AVE) values exceeded the acceptable threshold of 0.5, which confirmed the convergence validity of ILS. By comparing the square roots of AVE of all variables and their correlation coefficient with other variables, the results showed that the former was greater than the latter, which indicated that ILS had good discriminant validity and the five dimensions of Internet literacy were not difficult to distinguish. Specifically, the results of CFA demonstrated that KSI, ALI, ACI, II, ISM as five dimensions constitute the Internet literacy of Chinese youth and adolescents, with the details being presented in Table 3.

**Table 3:** Confirmatory factor analysis of Internet literacy.

Paths			Estimate	AVE	CR
Knowledge and skills for the Internet (KSI)					
KSI	--->	ILS-01	0.680	0.530	0.849
KSI	--->	ILS-02	0.686		
KSI	--->	ILS-03	0.767		
KSI	--->	ILS-04	0.744		
KSI	--->	ILS-05	0.758		
Autonomous learning on the Internet (ALI)					
ALI	--->	ILS-06	0.849	0.670	0.802
ALI	--->	ILS-07	0.787		
Awareness and cognition of Internet (ACI)					
ACI	--->	ILS-08	0.693	0.594	0.854
ACI	--->	ILS-09	0.774		
ACI	--->	ILS-10	0.842		
ACI	--->	ILS-11	0.767		
Internet interactions (II)					
II	--->	ILS-12	0.687	0.546	0.827
II	--->	ILS-13	0.661		
II	--->	ILS-14	0.801		
II	--->	ILS-15	0.797		
Internet self-management (ISM)					
ISM	--->	ILS-16	0.919	0.682	0.865
ISM	--->	ILS-17	0.806		
ISM	--->	ILS-18	0.743		

AVE is the abbreviation for average variance extracted values. CR is the abbreviation for composite reliability.

### 4.3. Estimation of the conceptual model

**Figure 2:**
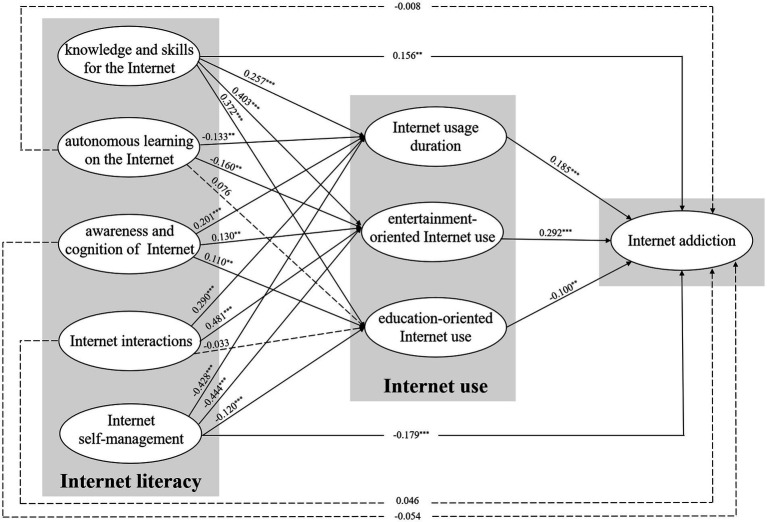
Path analysis of the structural model of the inter-relationships between Internet literacy, Internet use, and Internet addiction. *p < 0.05, **p < 0.01, ***p < 0.001, *N*=2276. The solid line represents the path is significant, while the dotted line represents the path is not significant. Model fit statistics: χ^2^/df=6.916, GFI=0.929, NFI=0.929, IFI=0.939, TLI=0.923, CFI=0.939, RMSEA=0.051.

Regarding the conceptual model developed founded on the literature review and proposed research questions, the SEM was adopted to explore the inter-relationships between Internet literacy, Internet use and Internet addiction among Chinese youth and adolescents. Figure 2 showed that all the fit indexes were completely in accord with acceptable standards, which fully indicated that the model had a good fitting effect.

As for RQ1, the results clearly illustrated that Internet usage duration (*β* = 0.185, *p* < 0.001) significantly and positively affected Internet addiction, while two types of IU influenced Internet addiction differently. To be specific, entertainment-oriented IU (*β* = 0.292, *p* < 0.001) affected Internet addiction significantly and positively, but education-oriented IU (*β* = −0.100, *p* < 0.01) affected Internet addiction negatively among Chinese youth and adolescents.

To answer RQ2, the analytical results suggested that KSI exerted a significant and positive effect on Internet usage duration (*β* = 0.257, *p* < 0.001), entertainment-oriented IU (*β* = 0.403, *p* < 0.001), and education-oriented IU (*β* = 0.372, *p* < 0.001), and the same applied to the impact of ACI on Internet usage duration (*β* = 0.201, *p* < 0.001), entertainment-oriented IU (*β* = 0.130, *p* < 0.01), and education-oriented IU (*β* = 0.110, *p* < 0.01). Besides, ALI had a significant and negative impact on Internet usage duration (*β* = −0.133, *p* < 0.01) and entertainment-oriented IU (*β* = −0.160, *p* < 0.01), while its impact on education-oriented IU (*β* = 0.076, *p >* 0.05) was not significant. Moreover, II affected Internet usage duration (*β* = 0.290, *p* < 0.001) and entertainment-oriented IU (*β* = 0.481, *p* < 0.001) positively, but did not have a significant impact on education-oriented IU (*β* = −0.033, *p >* 0.05). Finally, ISM had a significant and negative impact on Internet usage duration (*β* = −0.428, *p* < 0.001), entertainment-oriented IU (*β* = −0.444, *p* < 0.001), and education-oriented IU (*β* = −0.120, *p* < 0.001).

Regarding RQ3, the results indicated that KSI (*β* = 0.156, *p* < 0.01) influenced Internet addiction significantly and positively, while ISM (*β* = −0.179, *p* < 0.001) affected Internet addiction significantly and negatively. However, the effects of ALI (*β* = −0.008, *p >* 0.05), ACI (*β* = −0.054, *p >* 0.05), and II (*β* = 0.046, *p >* 0.05) on Internet addiction were not significant. The results of SEM were shown in Figure 2 in details.

## 5. Discussion and implications

In current digital age, Internet addiction has become a pressing issue among youth and adolescents in China. As digital native, today’s young generation rely on the Internet use for both entertainment and education, which was especially prominent during the COVID-19 pandemic. To examine the mechanism of Internet use on Internet addiction, and explore the role of multi-dimensional Internet literacy, this study investigated links between Internet literacy, Internet use and Internet addiction among Chinese youth and adolescents. Based on a systematic sampling survey conducted in a county-level city in East China and comprehensively measured Internet literacy and Internet use, this study captured more fine-grained aspects of Chinese adolescents’ digital life.

Consistent with previous studies, longer Internet usage duration can lead to Internet addiction among the young. Not surprisingly, Internet usage duration was closely linked to addictive Internet use, since the principal manifestation of Internet addiction is involuntary and long-term compulsive use of the Internet. However, based on the above findings, more attention should be paid to distinguish different patterns of Internet use, which may have varied impacts on Internet addiction. Specifically, the results demonstrated that more entertainment-oriented IU caused higher degree of Internet addiction, while more education-oriented IU was significantly correlated to lower degree of Internet addiction. As Young emphasized, the Internet itself is not addictive, it is vital to compare different types of Internet use and find out their influences on Internet addiction (9). As found in this study, youth and adolescents who used the Internet for entertainment or social interaction tended to show higher degree of Internet addiction, while those who engaged in education-oriented Internet activities such as information acquisition and online learning were more likely to have a lower degree of Internet addiction. Therefore, as shown in previous studies, prevention and intervention of Internet addiction should target those high risk groups who often unable to control their online time and related behaviors, and spent countless hours chatting, socializing or playing games on the Internet (28, 50).

As a multi-dimensional concept, the structure of Internet literacy was again confirmed in this research. As for Chinese youth and adolescents, Internet literacy can be divided into five sub-dimensions, i.e., KSI, ALI, ACI, II and ISM. Although there are some differences in conceptualization of Internet literacy, current concrete connotations are relatively consistent in a heuristic theoretical framework (51) that not only pays attention to knowledge learning and skill development, but also lays emphasis on the cultivation of social–emotional and critical literacy. In the present study, KSI is more focus on technical dimension, while ACI is close to cognitive dimension. As the social–emotional perspective of Internet literacy, II mainly evaluates the degree of interaction with others on the Internet, which is related to the sociability to communicate, collaborate, and handle daily routines online. ALI assesses the level of adolescents’ spontaneous learning through the Internet, and ISM measures self-control ability of youth and adolescents and whether they can manage their online time reasonably, both of which belong to critical literacy. It is worthy to note that Internet literacy as a comprehensive concept is not immutable and frozen, but needs to advance with the times and add new content or elements. For example, it is not uncommon for youth and adolescents to suffer from psychological illness caused by online risks such as cyberbullying and privacy disclosure, so Internet security and privacy literacy may become an important component of adolescents’ Internet literacy in the future.

Investigating Internet literacy as a comprehensive and exhaustive concept with five dimensions, this study further captured more precisely inter-relationships between Internet literacy and Internet use among Chinese youth and adolescents. This study found that higher levels of KSI and ACI positively correlated with a higher level of Internet use, including Internet usage duration, as well as both entertainment-oriented and education-oriented IU, and II associated with Internet usage duration and entertainment-oriented IU positively. However, adolescents with a higher degree of ISM were found to show less Internet use, and those with a higher degree of ALI showed lower levels of Internet usage duration and entertainment-oriented IU. In general, those youth and adolescents who had higher degree of KSI, ACI, and II tended to show more Internet use, while those who had higher degree of ISM and ALI turned out to show less Internet use, especially for Internet usage duration and entertainment-oriented IU. Thus, Internet literacy as a multi-dimensional concept plays a complicated role in affecting Internet use, with different dimensions showing even conflicting impacts.

Similarly, findings about the direct effects of Internet literacy on adolescent Internet addiction also suggest that not all types of Internet literacy will play a positive part in IA prevention and intervention. This study finds out that only ISM can effectively reduce adolescent Internet addiction, while KSI may actually increases Internet addiction. Consistent with previous research (27, 37, 42, 43), adolescents with higher self-management ability can be helpful to reduce Internet addiction, while the cultivation of KSI may require adolescents to devote a lot of time to study and practice on the Internet. Whereas, the more time youth and adolescents spend online, the higher possibility that they may develop Internet addiction. These findings mean that critical Internet literacy (especially ISM) can help prevent the development of Internet addiction among youth and adolescents, while functional Internet literacy (especially KSI) may increases the risk of Internet addiction.

Based on these findings, practical implications are provided. The first implication is embodied in the diagnosis of Internet addiction among youth and adolescents. In real life, many people relied on the amount of time took on the Internet or the frequency of Internet use to reflect adolescents’ excessive Internet use. However, due to its simplicity, time- and frequency-based measurements cannot take into account why adolescents use the Internet or what they do online (52). This study highlighted that it is necessary to combine Internet usage duration with specific types of Internet use into the diagnosis of Internet addiction among youth and adolescents, rather than relying solely on time or frequency as the single indicator. According to the findings of this study, if adolescents use the Internet for online learning for longer time, the probability of Internet addiction can even be greatly reduced. On the contrary, if adolescents cost plenty of time online for leisure and entertainment, it is essential for their family members, school teachers, community and health professionals to provide reasonable guidance for them to avoid them being addicted to the Internet. Those young Internet users with prolonged and more entertainment-oriented IU should be paid more attention for higher risks of Internet addiction. This study also contributes to prevention and intervention of Internet addiction for adolescents themselves, parents, clinicians, public health staff, and policy makers. For young Internet users, critical Internet literacy, especially ISM, should be improved to cope with Internet addiction, while functional Internet literacy, such as KSI, may have a hidden risk for forming Internet addiction. Therefore, more education and strategies should be done to nurture and improve adolescents’ critical Internet literacy in order to cope with the negative effects of Internet addiction, instead of simply teaching their Internet operational skills.

## 6. Limitations

Despite the above-mentioned findings, the current study still carries some limitations. First, due to the cross-sectional nature of the data, path analysis cannot guarantee casual relationships. Future research can examine the directionality of the association between Internet literacy and Internet addiction by using experimental or longitudinal designs. Second, although Internet use was systematically measured in this study to reflect the diversity of online activities and avoid the disadvantages of dichotomy measurement, the combination of self-reported measurement and objective methods would contribute to future studies (53), such as using smartphone applications or social media data to assess users’ Internet usage in more details. Third, given that inter-regional differences in the prevalence of Internet addiction in China and many countries around the world (5), there is a need to perform research in more culturally-varied settings to describe the cross-cultural aspects of the relationship between Internet literacy, Internet use, and Internet addiction. Lastly, Internet addiction as measured in general, while in the future, specific types of Internet addiction, such as social media addiction, smartphone addiction, gaming addiction, can be investigated to provide more understanding of the role that Internet literacy plays in specific type of Internet addiction among youth and adolescents.

## 7. Conclusion

To deal with the pressing issue of Internet addiction among the young in China, this study investigated the links between Internet literacy, Internet use and Internet addiction. Excessive Internet use and entertainment-oriented IU were found to closely correlate with Internet addiction among Chinese youth and adolescents, while education-oriented IU exerted negative effects on Internet addiction. As a multidimensional and comprehensive concept, ALI and ISM dimensions of Internet literacy reduced Internet use among youth and adolescents, while KSI, ACI, and II had exactly the opposite effect on Internet use. Not all sub-types of Internet literacy can reduce the degree of Internet addiction in youth and adolescents. Wherein, critical literacy (especially ISM) is helpful to cope with Internet addiction, while functional literacy (especially KSI) can be associated with higher risk for Internet addiction. Therefore, special emphasis should be placed on critical Internet literacy rather than functional literacy regarding prevention and intervention for Internet addiction targeting youth and adolescents.

## Data availability statement

The raw data supporting the conclusions of this article will be made available by the authors, without undue reservation.

## Ethics statement

The studies involving humans were approved by Tsinghua University Science and Technology Ethics Committee (Humanities, Social Sciences and Engineering). The studies were conducted in accordance with the local legislation and institutional requirements. Written informed consent for participation in this study was provided by the participants’ legal guardians/next of kin.

## Author contributions

QJ: conceptualization, methodology, supervision, writing – original draft, writing – review and editing, and funding acquisition. ZC: conceptualization, formal analysis, writing – original draft, and writing – review and editing. ZZ: investigation, software, and writing – review and editing. CZ: conceptualization, methodology, data curation, resources, validation, and project administration. All authors contributed to the article and approved the submitted version.

## Funding

This work was supported by the National Social Science Foundation of China (grant no. 20BXW127) and Tsinghua Lab Research Program on Computational Communication and Intelligent Media (grant no. 2023TSJCLAB001).

## Conflict of interest

The authors declare that the research was conducted in the absence of any commercial or financial relationships that could be construed as a potential conflict of interest.

## Publisher’s note

All claims expressed in this article are solely those of the authors and do not necessarily represent those of their affiliated organizations, or those of the publisher, the editors and the reviewers. Any product that may be evaluated in this article, or claim that may be made by its manufacturer, is not guaranteed or endorsed by the publisher.
