# Supplementary material for: Investigating links between Internet literacy, Internet use, and Internet addiction among Chinese youth and adolescents in the digital age
Source: Front Psychiatry. 2023 Sep 7;14:1233303. doi: 10.3389/fpsyt.2023.1233303 (PMC10513100; doi:10.3389/fpsyt.2023.1233303)
Supplement: Supplementary file 1 [file Data_Sheet_1.docx]

**Appendix 1. Internet use duration of the adolescents and youth in this study**

|  | **Weekdays** | | **Weekends or holidays** | |
| --- | --- | --- | --- | --- |
|  | Number | Proportion(%) | Number | Proportion(%) |
| **Not at all** | 1541 | 67.7 | 229 | 10.1 |
| **Within an hour** | 548 | 24.1 | 859 | 37.8 |
| **1 to 2 hours** | 119 | 5.2 | 636 | 27.9 |
| **2 to 3 hours** | 45 | 2 | 303 | 13.3 |
| **Over 3 hours** | 23 | 1 | 249 | 10.9 |
| **Total** | 2276 | 100 | 2276 | 100 |

**Appendix 2.** **The Internet literacy scale (Huang et al., 2021)**

| No. | Items | Scores | | | | |
| --- | --- | --- | --- | --- | --- | --- |
|  |  | 1 | 2 | 3 | 4 | 5 |
| 01 | I know how to solve technical problems in Internet usage. |  |  |  |  |  |
| 02 | I can make good use of Internet tools, such as office software and search engines. |  |  |  |  |  |
| 03 | I can get the information rapidly and accurately on the Internet. |  |  |  |  |  |
| 04 | I can express my opinions online through text, sound, images, etc. |  |  |  |  |  |
| 05 | I can use Internet resources creatively, such as searching for various materials to make PowerPoint. |  |  |  |  |  |
| 06 | I can access and analyze useful information on the website to complete learning tasks. |  |  |  |  |  |
| 07 | I can use the Internet to improve myself, such as learning meaningful knowledge and skills. |  |  |  |  |  |
| 08 | We should respect different Internet cultures in the world. |  |  |  |  |  |
| 09 | We should discern harmful information while surfing the Internet. |  |  |  |  |  |
| 10 | I think the Internet is a double-edged sword. |  |  |  |  |  |
| 11 | Faced with different opinions on the Internet, we should communicate rationally with each other. |  |  |  |  |  |
| 12 | I am able to make new friends through the Internet. |  |  |  |  |  |
| 13 | I can show myself through social media, such as WeChat, QQ, and TikTok. |  |  |  |  |  |
| 14 | The Internet enables me to better collaborate with my friends in daily activities. |  |  |  |  |  |
| 15 | I often get help from my friends via the Internet. |  |  |  |  |  |
| 16 | When I study online, I am not easily attracted by other irrelevant information. |  |  |  |  |  |
| 17 | I can control how much time I spend online. |  |  |  |  |  |
| 18 | I have a plan when I am surfing the Internet. |  |  |  |  |  |

**Appendix 3. The results of parallel analysis**

| **Root** | **Row Data** | **Means** | **Prcntyle** |
| --- | --- | --- | --- |
| 1 | 1.991 | 1.046 | 1.072 |
| 2 | 1.039 | 1.013 | 1.031 |
| 3 | 0.529 | 0.987 | 1.000 |
| 4 | 0.441 | 0.954 | 0.977 |

**Appendix 4. Path analysis of the structural model of the inter-relationships between Internet literacy, Internet use and Internet addiction among the subgroup of primary school students**


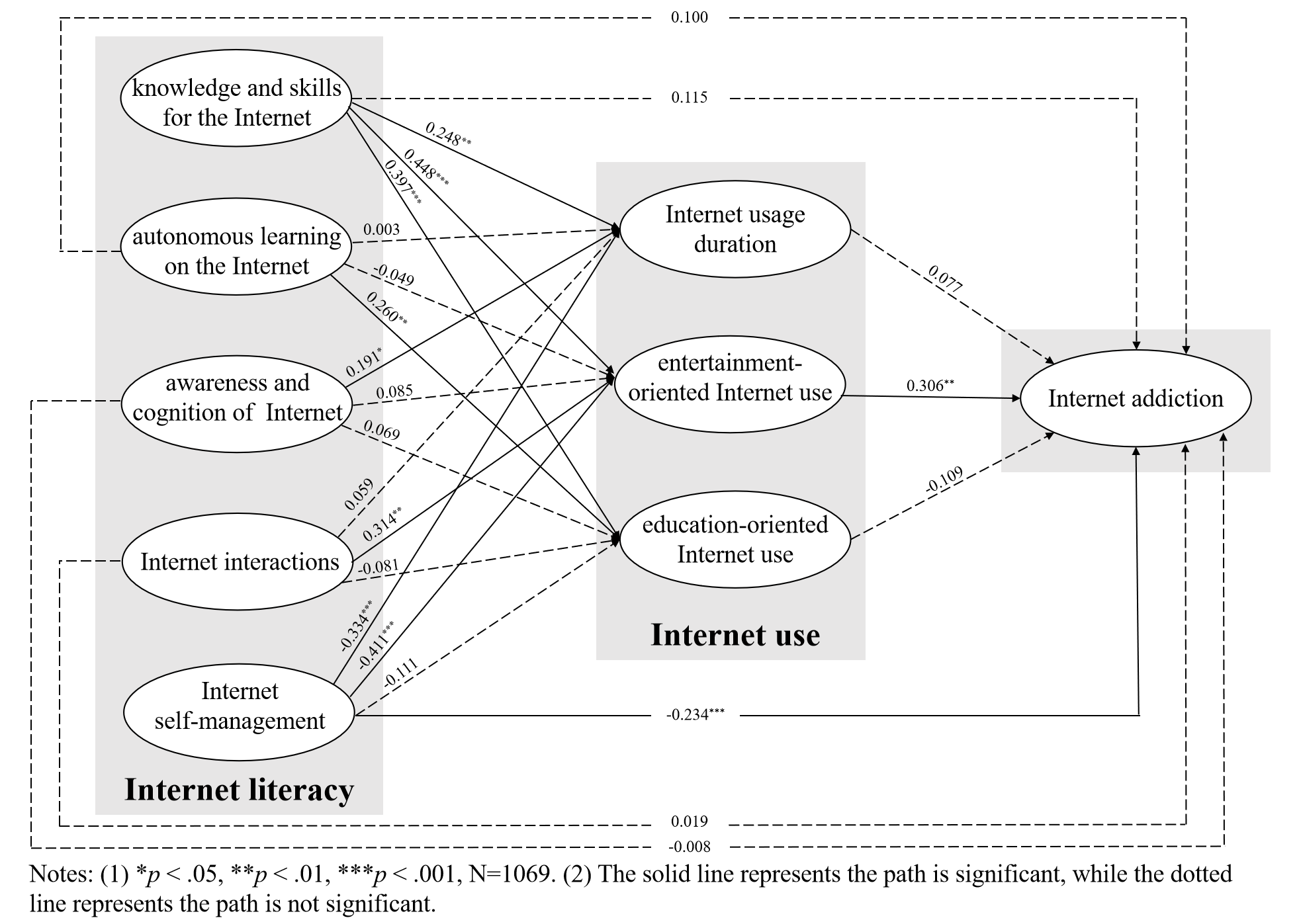


**Appendix 5. Path analysis of the structural model of the inter-relationships between Internet literacy, Internet use and Internet addiction among the subgroup of secondary school students**


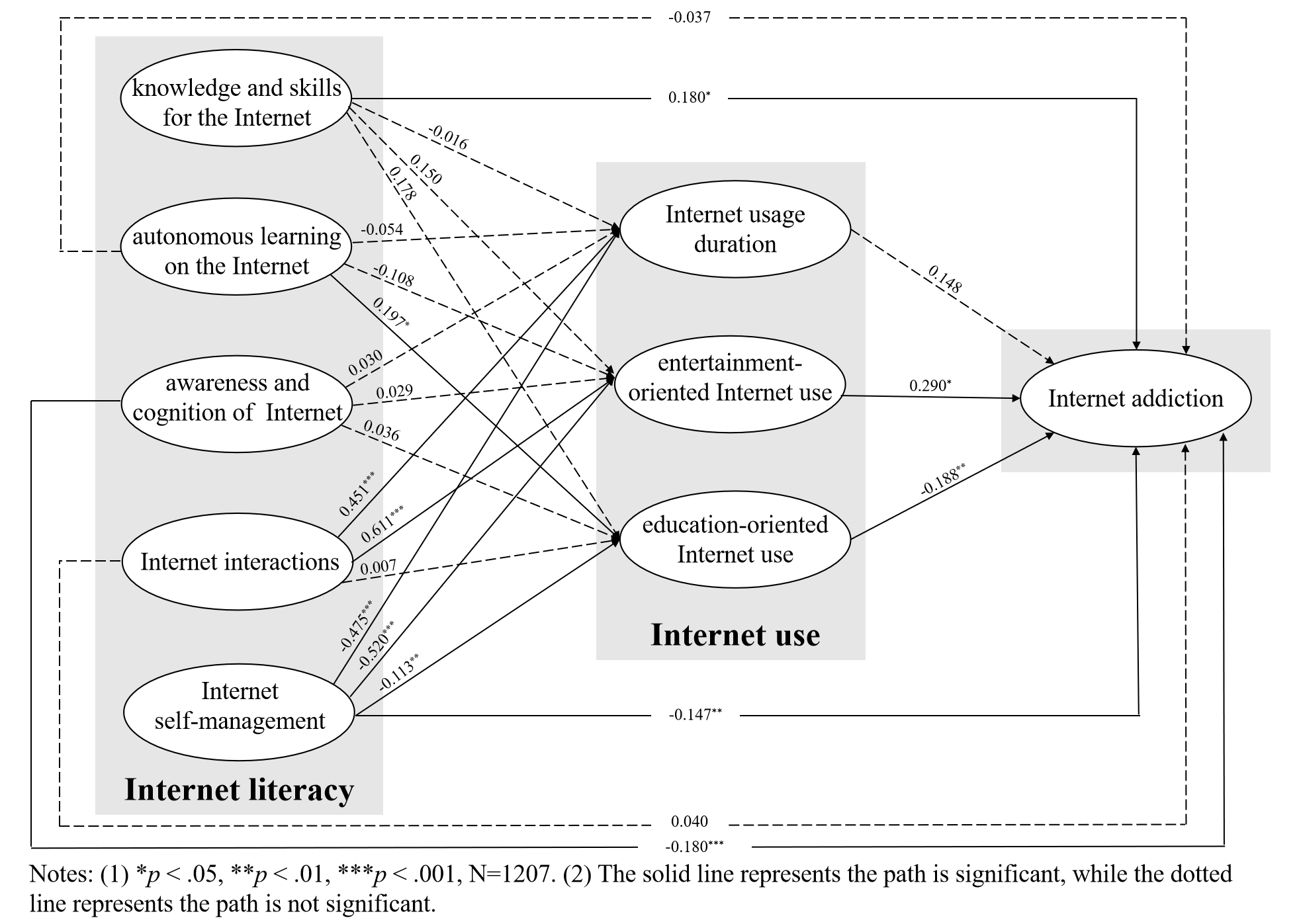


**Appendix 6. The main fit statistics of the four different models**

| **Model** | **χ^2^/df** | **GFI** | **NFI** | **IFI** | **TLI** | **CFI** | **RMSEA** |
| --- | --- | --- | --- | --- | --- | --- | --- |
| **Unconstrained** | 4.223 | 0.915 | 0.908 | 0.928 | 0.910 | 0.928 | 0.038 |
| **Measurement weights** | 4.319 | 0.911 | 0.903 | 0.924 | 0.907 | 0.924 | 0.038 |
| **Structural weights** | 4.346 | 0.907 | 0.900 | 0.921 | 0.906 | 0.921 | 0.038 |
| **Structural covariances** | 4.594 | 0.899 | 0.892 | 0.914 | 0.899 | 0.913 | 0.041 |

**Appendix 7. The increase in NFI, IFI, RFI, and TLI under the assumption of model unconstrained to be correct.**

| **Model** | **NFI Delta-1** | **IFI Delta-2** | **RFI rho-1** | **TLI rho-2** |
| --- | --- | --- | --- | --- |
| **Measurement weights** | .005 | .005 | .003 | .003 |
| **Structural weights** | .008 | .008 | .003 | .003 |
| **Structural covariances** | .016 | .016 | .010 | .010 |
